# Increasing incidence of lung transplantation in patients with post-COVID-19 pulmonary fibrosis

**DOI:** 10.11604/pamj.2021.40.169.31223

**Published:** 2021-11-18

**Authors:** Rumi Khajotia

**Affiliations:** 1 Department of Internal Medicine, International Medical University, Seremban, Malaysia

**Keywords:** Lung transplantation, thoracic Surgery, COVID-19 infection, pulmonary fibrosis, pulmonary medicine, interstitial damage

## Abstract

Twenty months into the COVID-19 pandemic, we are still learning about the various long-term consequences of COVID-19 infection. While many patients do recover with minimal long-term consequences, some patients develop irreversible parenchymal and interstitial lung damage leading to diffuse pulmonary fibrosis. Unfortunately, these are some of the consequences of post-SARS-CoV-2 infection which thousands more people around the world will experience and which will outlast the pandemic for a long time to come. It is now being observed at various leading medical centres around the world that lung transplantation may be the only meaningful treatment available to a select group of patients experiencing serious lung damage and non-resolving COVID-19-associated respiratory failure, resulting from the triad of coronavirus infection, a hyper-inflammatory immune response to it and the inability of the human body to repair that injury.

## Commentary

One of the most common long-term complications seen in patients who have recovered from COVID-19 infection include severe parenchymal and interstitial lung damage resulting in the development of pulmonary fibrosis. These long-term complications in COVID-19 patients are believed to be due to various factors such as SARS-CoV-2 induced viral toxicity, capillary thrombosis, acute respiratory distress syndrome (ARDS), ventilator-associated lung injury, drug toxicity and the use of extracorporeal membrane oxygenation (ECMO) during the course of their initial illness. The COVID-19 infection and the hyper-immune response causing a cytokine storm finally result in the development of honeycombing and thick fibrotic scar tissue, sometimes within a matter of weeks of the COVID-19 infection, creating extensive honeycomb changes that make the lungs completely stiff and solid, destroying the normal alveolar sacs and the interstitium. This affects normal gas exchange. Consequently, patient survival hinges on intubation and ventilatory management, in such cases.

This has resulted in an increasing incidence of lung transplantations around the world in order to improve patient survival, which would otherwise be impossible, considering the extent of lung tissue damage in these post-COVID-19 infection patients. At Northwestern Memorial Hospital, Chicago, IL, USA [1], three lung transplantations were performed in patients with respiratory failure following coronavirus-induced pneumonia. All three patients suffered extensive lung injury requiring ECMO in order to maintain adequate oxygenation. In all these patients the lungs showed multiple incidence of ventilator-associated pneumonia, repeated episodes of pneumothorax requiring intercostal drainage, hemothorax and consequently a significant reduction in lung compliance. The pathological lung findings in all three patients showed extensive lung injury with large areas of bronchopneumonia and diffuse alveolar damage, accompanied by extensive pleural inflammation and bleeding into the alveolar spaces. In addition, the pathological findings in the lungs of two patients showed multiple lung cysts bilaterally. The surrounding interstitium also showed significant thickening and fibrosis with significant loss of alveolar architecture, in both these patients. These pathological features were believed to be due to extensive lung injury from coronavirus-induced pneumonia accompanied by ventilator-induced lung damage and extensive thrombosis.

All three patients who underwent lung transplantation in the above study were given a three-drug immunosuppression regimen post-surgery, which included steroids, anti-metabolite and calcineurin inhibitors. In addition, they were given solumedrol and basiliximab before reperfusion, as part of the induction immunosuppression protocol for lung transplants. In another early study [2] conducted between February´20-March´20, three male patients with COVID-19 underwent lung transplantation. All three patients had illness duration of more than one month with very high sequential organ failure assessment scores. Of these, two patients survived post-transplantation and were able to actively participate in the rehabilitation program. The pathology of the explanted lungs was concordant with the critical clinical manifestation and provided better understanding of the disease process. This early

Study suggested that successful lung transplantations can be performed in a select group of patients with severe respiratory distress and failure due to COVID-19-related pulmonary fibrosis. In another larger international study[3] conducted between 1^st^ May´20 and 30^th^ September´20, a total of twelve patients with ARDS following COVID-19 infection, who had undergone bilateral lung transplantation, were studied. These included eight patients at medical centres across the USA, two patients in Italy, one patient in Austria and one in India. The median age of the lung transplant patients was 48 years and three of them were female. Chest computed tomography (CT)-scans showed diffuse lung injury which persisted inspite of extensive mechanical ventilation and extracorporeal membrane oxygenation (ECMO). The surgery was challenging in these patients as they had extensive hilar lymphadenopathy and pleural adhesions which consequently increased intraoperative blood transfusion requirements due to bleeding from the vascular adhesions. Pathology of the affected lungs showed extensive lung fibrosis. Fortunately, there was no recurrence of the coronavirus infection in the allografts. Following surgery, all patients were successfully weaned off ECMO support. In the above case series, the patient admitted to the Medical University of Vienna, Austria [3,4] had severe pulmonary fibrosis following COVID-19 infection. After nearly two months of medical treatment a donor organ was available and bilateral lung transplantation was carried out. During the surgery, a venoarterial extracorporeal membrane oxygenation circuit was installed. Due to widespread lung destruction, the normal lung anatomy was grossly altered. As a result, only extrapleural mobilisation of the lungs could be carried out. The implantation was also difficult as the patient´s bronchus and vessels were very fragile and vascular due to inflammatory changes caused by the COVID-19 infection. Even though extensive haemostasis was performed, a total of thirty units of packed red blood cells and five units of platelets were needed to be given to the patient during the lung transplant procedure. Post-operatively, the patient was kept in the ICU. Thereafter, the patient showed good recovery and the venoarterial ECMO was removed on the third day following surgery. The grading for primary graft dysfunction was zero at 72 hours post-surgery. The patient was further administered standard triple immunosuppression, including tacrolimus, mycophenolate mofetil, and steroids.

While brain-dead donors are most commonly used for full lung transplants in post-COVID-19 patients, the first live donor double-lung transplant was conducted at Kyoto University Hospital in Japan, in April 2021 [5]. The patient, a woman from the Kansai region in western Japan had suffered coronavirus infection-induced pneumonia followed by severe bilateral lung damage, which resulted in her requiring extracorporeal membrane oxygenation (ECMO). Both her lungs had become severely non-functional and stiff and her doctors decided that the only way she could survive was to have a double-lung transplant. Both her husband and her son donated parts of their lungs for her surgery. Consequently, both her lungs were removed and she received part of her son´s right lung and a part of her husband´s left lung. The entire surgery took 10 hours and 57 minutes. Following the surgery, the patient successfully came off the ECMO but needed ventilator therapy for some period of time. These early extensive studies done internationally indicate the absolute usefulness of lung transplantation in select patients with severe post-COVID-19 pulmonary fibrosis who have extensive lung damage impairing gas exchange, thereby resulting in severe breathlessness and respiratory failure.

From the above studies it is evident that lung transplantations are pivotal to the survival of many patients infected with SARS-CoV-2, who have subsequently developed severe lung injury and extensive pulmonary fibrosis. However, lung transplantations are not without limitations. These include a resurgence of the coronavirus infection in the transplanted allograft, surgical difficulties imposed by the virus-induced injury to the patient´s lung, and the risk of the allograft getting infected by pathogenic organisms causing ventilator-associated pneumonia in the native lung. It is also important to remember that since SARS-CoV-2 predominantly invades the respiratory tract, in case of lung transplantation there are also limitations and concerns regarding donor-derived viral transmission and impaired quality of graft [6]. Hence, for now the learning curve is particularly steep for lung transplantations in post-COVID-19 patients, since the recipient´s immune system needs to be suppressed adequately in order to reduce the chance of organ rejection and allow the donor lung to survive post- transplant.

However, due to their immunocompromised status, such patients could be further susceptible to coronavirus infection in the already fragile transplanted lung. Consequently, ‘rejection-infection’ need to be counter-balanced in order to achieve success post-lung transplantation, in these patients. In case of post-COVID-19 lung transplantation, it is also very important that the procedure be timed properly. If the lung transplantation is done too soon there is a chance that the patient is not yet completely free of his coronavirus infection and could still be harbouring the virus and thereby develop a relapse of infection later. Also, if the lung transplantation is done too late it may not be beneficial as the patient may already be in a deteriorated condition with severely compromised lung function, pulmonary hypertension and right-sided heart failure. In such cases a simultaneous heart-lung transplant may then become necessary. There is now a general belief that in patients younger than age 65, lung transplants should be done at least four-to-six weeks following the onset of COVID-19 infection when there is evidence of end-stage lung damage. This is because there is general consensus that patients should be conscious enough to participate in their decision-making process and be overall healthy enough to engage in the rehabilitation process following the transplant surgery.

As yet, since we are only 20 months into the pandemic, most of the post-SARS-CoV-2 related lung transplants have involved patients with acute lung damage. However, with the passage of time, it is obvious that a new class of patients will arise as a consequence of the long drawn-out pandemic; namely those patients who now develop chronic lung damage as a result of the infection. With continuing advances in surgical techniques and post-operative management of COVID-19 patients, there are also now reports of multiple procedures being carried out simultaneously alongside lung transplantation, in these patients. A recent report from New York-Presbyterian/Columbia University stated that a post-SARS-CoV-2 infection patient had recently undergone a simultaneous lung transplantation and triple-vessel coronary artery bypass graft there.

In conclusion, while lung transplantation is by no means a treatment option for every patient infected with SARS-CoV-2, in some patients lung transplantation certainly helps liberate patients from the acute care setting, which would otherwise have been impossible due to severely damaged lungs. Lung transplantation has proved to be a life-saving procedure in some SARS-CoV-2 infected patients whose lungs have been irreversibly damaged by the virus, resulting in widespread pulmonary fibrosis and consequent severe respiratory distress and failure.

## Conclusion

It is now generally accepted that a potential lung transplantation should be considered in a patient below age 65 years who has no significant comorbidities and is in otherwise good physical condition, in whom all other medical treatment options have been exhausted, there is significant, irreversible damage to the lungs on repeated chest-CT scans and in whom no significant recovery is observed in the post-SARS-CoV-2 infected lungs following at least 4 weeks of mechanical ventilation or ECMO.
